# Estimating the Incidence of Conjunctivitis by Comparing the Frequency of Google Search Terms With Clinical Data: Retrospective Study

**DOI:** 10.2196/22645

**Published:** 2021-03-03

**Authors:** Paola Kammrath Betancor, Linda Tizek, Alexander Zink, Thomas Reinhard, Daniel Böhringer

**Affiliations:** 1 Eye Center, Medical Center Faculty of Medicine University of Freiburg Freiburg Germany; 2 Department of Dermatology and Allergy School of Medicine Technical University of Munich Munich Germany

**Keywords:** epidemic keratoconjunctivitis, big data, Google search, Freiburg clinical data

## Abstract

**Background:**

Infectious conjunctivitis is contagious and may lead to an outbreak. Prevention systems can help to avoid an outbreak.

**Objective:**

We aimed to evaluate if Google search data on conjunctivitis and associated terms can be used to estimate the incidence and if the data can provide an estimation for outbreaks.

**Methods:**

We obtained Google search data over 4 years for the German term for conjunctivitis (“Bindehautentzündung”) and 714 associated terms in 12 selected German cities and Germany as a whole using the Google AdWords Keyword Planner. The search volume from Freiburg was correlated with clinical data from the Freiburg emergency practice (Eye Center University of Freiburg).

**Results:**

The search volume for the German term for conjunctivitis in Germany as a whole and in the 12 German cities showed a highly uniform seasonal pattern. Cross-correlation between the temporal search frequencies in Germany as a whole and the 12 selected cities was high without any lag. Cross-correlation of the search volume in Freiburg with the frequency of conjunctivitis (International Statistical Classification of Diseases and Related Health Problems [ICD] code group “H10.-”) from the centralized ophthalmologic emergency practice in Freiburg revealed a considerable temporal association, with the emergency practice lagging behind the frequency. Additionally, Pearson correlation between the count of patients per month and the count of searches per month in Freiburg was statistically significant (*P*=.04).

**Conclusions:**

We observed a close correlation between the Google search volume for the signs and symptoms of conjunctivitis and the frequency of patients with a congruent diagnosis in the Freiburg region. Regional deviations from the nationwide average search volume may therefore indicate a regional outbreak of infectious conjunctivitis.

## Introduction

Conjunctivitis is a very common diagnosis. Viral conjunctivitis is one of the most frequent eye infections worldwide [1]. It may be extremely contagious and often begins with very uncharacteristic symptoms. They include redness, photophobia, watery eyes, and foreign body sensation. Patients can be infectious even days before having any symptoms and weeks after symptoms have improved [1]. The differential diagnosis of acute infectious conjunctivitis is difficult, since the signs and symptoms of other inflammatory conjunctival conditions are quite similar [2]. The common differential diagnoses are seasonal allergic conjunctivitis and bacterial conjunctivitis. However, adenoviral keratoconjunctivitis is the most important disease because every infection may lead to keratitis nummularis. Hereby, subepithelial infiltrates can cause a reduction in the visual acuity and increase sensitivity to glare [3].

The correct diagnosis is important because infectious patients must be given sick leave to prevent spread, and the differential diagnoses need to be handled differently. This should be managed by an ophthalmologist because of the huge personal and socioeconomic impacts. The economic significance of epidemic keratoconjunctivitis has been quantified in some studies [4], but is very difficult to assess, as there is no reliable basis for a robust estimation of the affected population [5].

Since 2001, in Germany, adenoviral epidemic keratoconjunctivitis has been a notifiable disease according to the German Protection Against Infection Act (IfSG). If adenovirus is detected, the detecting laboratory (not the ophthalmologist) is obliged to report it. Only if the ophthalmologist has an urgent suspicion of an outbreak in his/her practice or hospital, he/she has the obligation to report it to the public health department, regardless of the pathogen [6]. Nevertheless, it can happen that the ophthalmologist diagnoses the disease on the basis of clinical symptoms alone. Therefore, it is possible that there is high underreporting. In addition, it can be assumed that a large proportion of adenovirus diseases are mild and self-healing and that these patients do not visit an ophthalmologist. From an epidemiological point of view, this is a serious problem because this precludes preventive measures to contain the outbreak [5].

Nowadays, the internet is one of the most used sources of health information. Patients “Google” their symptoms before seeing any doctor and may even end up without seeking professional health care at all [7]. Interestingly, infectious disease activity can be predicted by the frequency of internet searches. Studies related to influenza surveillance already proved that search-term surveillance is a possible supplement for disease-surveillance systems [8,9], whereas other studies showed a correlation of search data and cancer registries [10,11] or identified unmet medical needs in pruritus [12,13]. Internet biosurveillance systems use data from multiple sources. They refer to aggregator news sites like Google, social media sources like Facebook, text-based news sites, and apps [14].

The purpose of this study was to evaluate the potential of the analysis of Google search data to estimate the incidence of epidemic conjunctivitis. Therefore, we examined the relationship between searches for the German term for conjunctivitis (“Bindehautentzündung”) as well as 714 associated terms and actual conjunctivitis occurrence in the emergency practice of Freiburg over 4 years.

## Methods

### Data Collection

A retrospective longitudinal study using Google AdWords Keyword Planner was carried out to analyze the temporal course of keywords related to the German term for conjunctivitis (“Bindehautentzündung”) across Germany as well as across 12 German cities (Berlin, Hamburg, Munich, Cologne, Frankfurt [Main], Stuttgart, Hannover, Nuremberg, Freiburg, Leipzig, Rostock, and Kassel). Most of the cities were chosen because they are Germany’s largest cities by population (decreasing order: Berlin, Hamburg, Munich, Cologne, Frankfurt, Stuttgart, Leipzig, Hannover, and Nuremberg). Furthermore, Rostock was chosen to examine whether proximity to the coast has an influence on Google search volume. The remaining cities (Freiburg and Kassel) were chosen because a nationwide overview of various regions should be generated, and they are some of the largest cities within these regions. Primarily, the Keyword Planner is used for optimizing marketing campaigns; however, in recent years, it is being used to answer scientific questions. To examine the search volume for a specific topic, words or phrases are entered in the tool, and then, the tool finds the most relevant search terms with their average monthly search volume estimated by Google. In this study, the German term for conjunctivitis (“Bindehautentzündung”) was used to assess the search volume from November 2015 to October 2019. The region and language settings were set so that the Google data were limited solely to users across Germany whose language preference was German. The program identifies all keywords it considers relevant. The tool generally provides all the associated keywords for each city for an easier comparison, but this does not mean that in every city, people use the respective keywords. In our study, all 714 terms were used by people living in Berlin. The numbers of missing terms in the other cities were as follows: Hamburg, 4; Munich, 2; Cologne, 3; Frankfurt (Main), 13; Stuttgart, 9; Leipzig, 24; Hannover, 30; Nuremberg, 27; Freiburg, 90; Rostock, 136; and Kassel, 99. These missing search terms were imputed as 0. We eventually normalized the results per 100,000 inhabitants to account for differences in population size. The numbers of inhabitants in the German cities are provided in Multimedia Appendix 1.

We correlated the search frequencies with the actual patient frequencies on the basis of ICD (International Statistical Classification of Diseases and Related Health Problems) coding for the patients in Freiburg using data from the centralized ophthalmologic emergency practice for the Freiburg region. This anonymized data set, including postal codes, ICD codes, and date of presentation, was kindly provided by the Association of Statutory Health Insurance Physicians of Südbaden (“Kassenärztliche Vereinigung Südbaden”). The ICD codes of all patient visits from November 2015 to October 2019 were searched for the ICD “H10.-” code group (it is primarily for “conjunctivitis”, but also includes “mucopurulent conjunctivitis,” “acute allergic conjunctivitis,” “other acute conjunctivitis,” “not specified acute conjunctivitis,” “chronic conjunctivitis,” “blepharoconjunctivitis,” “other conjunctivitis,” and “not specified conjunctivitis”). In this time period, 28,775 patients visited the emergency practice. Of these, 3882 patients were diagnosed with a code from the ICD “H10.-” code group.

### Data Presentation and Statistical Analysis

We performed cross-correlations of the Google search data of Germany in total with the individual German cities. This was done to measure the regional variations of search frequencies for conjunctivitis within Germany. In the second step, we performed a cross-correlation between the search frequencies for Freiburg and the frequencies of the codes in the ICD “H10.-” code group from the centralized ophthalmologic emergency practice for the Freiburg region. This was done to estimate the association between search frequencies and frequencies of actual “H10.-” diagnoses. To achieve this, we had to interpolate the monthly search frequencies to obtain data at day resolution for the sum of all 714 associated terms. Statistical analysis was based on the R platform [15], using the function “qplot” (package ggplot2). For cross-correlation, we used “ccr” (base). Cross-correlation is a method to analyze similarities between two time series. This method provides a degree of similarity and a time lag that would be needed to align the two time series for optimal congruence. For Pearson correlation, we used “cor.test” (base). Pearson correlation assesses the strength of a linear association between two continuous variables. A *P* value <.05 was considered statistically significant [16].

## Results

**Figure 1:**
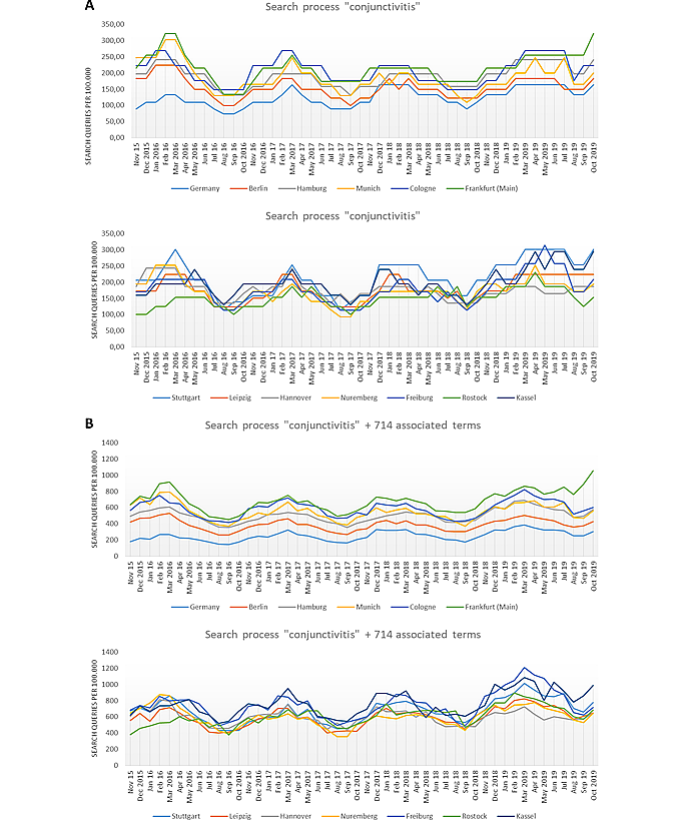
Search process for the German term for conjunctivitis (“Bindehautentzündung”) and 714 associated terms. (A) Search history of the term “conjunctivitis” from November 2015 to October 2019 in Germany and in the cities Berlin, Hamburg, Munich, Cologne, Frankfurt (Main), Stuttgart, Leipzig, Hannover, Nuremberg, Freiburg, Rostock, and Kassel per 100,000 inhabitants. (B) Same representation as (A) but for the search process of the term “conjunctivitis” and the 714 associated terms.

**Figure 2:**
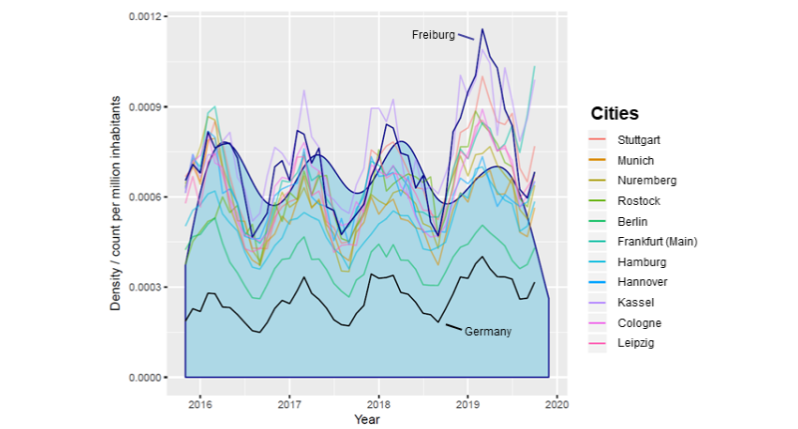
Density plot. Patients diagnosed with conjunctivitis (ICD code group "H10.-") in the centralized ophthalmologic emergency practice for the Freiburg region (light blue) superimposed with normalized cumulative Google search frequencies of all 714 terms for conjunctivitis. All curves show the same recurrent seasonal trend.

Google search data for the German term for conjunctivitis (“Bindehautentzündung”) from Germany and 12 German cities showed similar peaks of search queries per 100,000 inhabitants (Figure 1A and B). The search volume for the German term for conjunctivitis (“Bindehautentzündung”) in Germany totaled 5963 searches per 100,000 inhabitants, and in Freiburg, the search volume reached 8609 searches per 100,000 inhabitants. Since the general population tended to use a broad spectrum of layman’s terms for the symptoms of conjunctivitis, we repeated the procedure with 714 associated terms related to conjunctivitis that had been identified by Google Keyword Planner (Multimedia Appendix 2). The search volume in Germany as a whole was lower than in the individual cities (Figure 2). Including all the associated terms, the search volumes were 12,132 searches per 100,000 inhabitants in Germany and 35,926 searches per 100,000 inhabitants in Freiburg. In the 12 cities, the cumulative average search volume of all associated terms per 100,000 inhabitants was 240% higher in comparison to that for Germany as a whole. This hints toward lesser usage of Google in rural areas in comparison with the bigger cities included in our analysis. The curves for the search frequency of all associated terms showed a clear seasonal pattern (Figure 1). Cross-correlation between the temporal search frequencies in Germany as a whole and the 12 selected cities was generally high. The lowest cross-correlation coefficient (*r*) was 0.6 in Nuremberg and 0.8 for all other cities. This indicates a nationwide seasonal trend.

From 2015 to 2019, the centralized ophthalmologic emergency practice for the Freiburg region (“KV-Notfallpraxis Freiburg”) treated on average 5755 patients per year. Of these, an average of 776 had conjunctivitis every year. The demographic analysis of the patients diagnosed with ICD codes from the “H10.-” ICD group revealed a strong predominance of place of residence in Freiburg and its surroundings (data not shown). On average, 48% of all conjunctivitis cases belonged to Freiburg and its surroundings.

**Figure 3:**
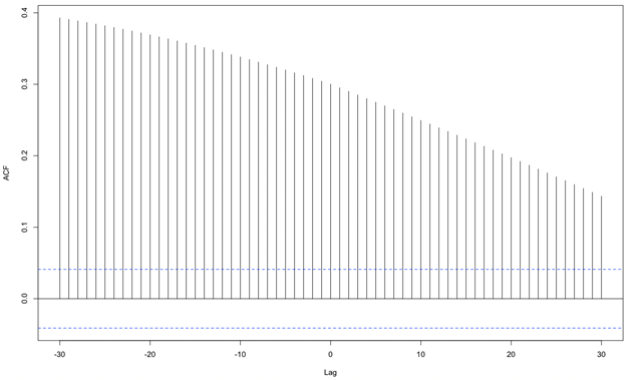
Cross-correlation between Google searches in Freiburg and the density of patients diagnosed with conjunctivitis (ICD code group "H10.-") in the Freiburg region. The visit density in the emergency practice lags behind the Google search. Each vertical bar corresponds to a bivariate correlation of the values at interest between both time series, with the second series shifted for the given time lag (given in days). The blue line depicts the upper limit of the correlation that one would see if one of the functions was only white noise [17]. The search frequencies are available only aggregated per month. Therefore, we had to intrapolate these data at day level before applying the cross-correlation function. ACF: autocorrelation function.

The cumulative Google search frequency of all associated terms in Freiburg revealed a temporal correlation with the density of patients diagnosed with conjunctivitis (ICD “H10.-”) in the centralized ophthalmologic emergency practice for the Freiburg region. The time lag between both time series was 30 days. The peak correlation coefficient was 0.39 (Figure 3). In this analysis, values higher than 0.04 have to be considered statistically significant as values ±0.04 are the extrema at an alpha level of .05 when one of the functions contains only white noise [17].

Pearson correlation between the count of patients per month from the centralized ophthalmologic emergency practice for the Freiburg region and the corresponding count of searches per month yielded a correlation coefficient of 0.3 (*P*=.04).

## Discussion

### Principal Findings

The longitudinal cumulative quantity of the Google search volume for all relevant keywords related to the German term for conjunctivitis (“Bindehautentzündung”) was roughly the same in Germany as a whole and in 12 large German cities in relation to the respective inhabitants. Since there was a clear temporal correlation between the cumulative Google search frequency of all 714 associated terms in Freiburg and the density of patients identified with codes in the ICD “H10.-” code group from the centralized ophthalmologic emergency practice for the Freiburg region, and the cross-correlation between the temporal search frequencies in Germany and the 12 selected cities was high, the clinical results from the Freiburg data can most likely be extrapolated to Germany as a whole.

It seems likely that the periodicity of the curves is caused by a seasonal effect since the timing of the highest search rates through the analyzed 4 years was very similar in the 13 curves (Figure 1). Seasonality is well known for infectious conjunctivitis [18]. This seasonality is also observable in the density plot for the patients diagnosed with conjunctivitis (ICD codes in the “H10.-” code group) in the centralized ophthalmologic emergency practice for the Freiburg region (Figure 2). It has to be considered that the ICD code group “H10.-” is used for not only viral or adenoviral conjunctivitis but also “acute allergic conjunctivitis.” Allergic conjunctivitis is frequently observed in spring and summer [19]. It is plausible to suppose that most patients with allergic conjunctivitis do not visit any ophthalmologist or consult the internet because they are familiar with the symptoms that recur each year. Since there is only one climate zone in Germany, the frequency of allergic conjunctivitis should be more or less the same all over the country. The seasonal patterns from Figure 1 may therefore be considered the “normal” background reference for Germany for consulting the internet for the signs and symptoms of all kinds of conjunctivitis. We hypothesize that a small fraction of patients with more severe or more protracted symptoms is more likely to actually see an ophthalmologist. This may explain why cross-correlation between the search frequency in Freiburg and the patient density revealed a substantial time lag between both curves (Figure 3).

Social media are nowadays some of the most common forms of electronic communication and are used to obtain information. However, potential sources of bias need to be considered. First, the restriction to only use data from a single commercial internet search engine may be problematic. However, since Google has an estimated market share of 95% in Germany, this restriction is acceptable at the time of our analysis [20]. Second, people who seek health information on the internet may not be a fully representative subset of the general population. Our data hint in that direction. Interestingly, the normalized search volume in Germany as a whole was much lower than that in the larger cities. This may have two reasons. First, Google usage may be generally higher in urban regions than in the countryside. Rural inhabitants may see an ophthalmologist directly without consulting online media. Second, rural inhabitants may be less concerned about their ocular health than urban inhabitants. They may ignore the signs and symptoms of conjunctivitis instead of exploring online media for information. Nevertheless, seasonal periodicity was clearly visible in the curve for Germany as a whole, and the correlation with individual cities was generally high without any time lag (Figure 2).

Bias is a bigger issue when active usage of the internet is analyzed, as was noted in a recent related analysis that evaluated social media posts [21]. They were machine classified as related to infectious conjunctivitis. Nevertheless, Deiner et al observed a similar correlation to clinical infectious cases and discussed their method as a possible way to improve the detection of outbreaks [21].

In addition to bias, potential sources of fuzziness of information need to be considered both in the usage of social media posts and analysis of Google. The timing people search for information is potentially not well defined. The search can occur without individuals having any symptoms because someone they know has symptoms, after seeing the doctor, or after having passed the infection. Additionally, the location of the search is extremely variable, since mobile devices give access to the internet nearly everywhere, even when traveling. However, owing to the large amount of data, the concept of detecting regional outbreaks via Google search volume analysis may work despite these sources of error.

There is a strong unmet need to improve the attention of authorities to outbreaks of contagious conjunctivitis to keep them in check. One approach is to improve office tests for adenovirus and tests in patients with severe bilateral conjunctivitis to increase sensitivity and specificity [22]. However, even if all patients with adenovirus conjunctivitis are detected this way, it only leads to diagnosis in the cohort of patients who seek professional health care. In addition, large studies have shown that a substantial proportion of keratoconjunctivitis does not appear to be associated with detectable adenovirus [23]. Moreover, we still have the problem of all those patients who do not visit an ophthalmologist and may be sources of infection for others.

Interestingly, all German cities in our data set showed a more or less synchronous pattern of search frequencies over the observation period. This could mean that this pattern is the “normal” background activity in the absence of an outbreak of epidemic keratoconjunctivitis. It could therefore be possible to automatically detect regional outbreaks from Google search data if regional search volumes behave differently from the national average or other reference regions. However, it is not clear at this point whether such an approach would reach appropriate specificity and sensitivity.

This has been discussed by Wilson et al, along with the advantages and disadvantages of internet-based outbreak surveillance. Looking at the advantages, they pointed out earlier detection, free access to data, and the potential to allow the public to have access to health surveillance information [24]. Another issue raised involves privacy. The Google data generated via the Keyword Planner cannot be attributed to age or gender. For this reason, the data are fully anonymous and cannot be reidentified using secondary data sets. However, it must be taken into consideration that certain restrictions on freedom may result in the case of a regional outbreak. People should be made aware if their search history is being used to generate surveillance data by competent authorities, even if the data are fully anonymous and reidentification is impossible.

In summary, our analysis demonstrates a clear correlation between the Google search volume for the signs and symptoms of conjunctivitis and the density of patients who present with the signs and symptoms of conjunctivitis. Furthermore, we characterize a seasonal pattern of the Google search volume in Germany that may serve as a “normal” background reference. With these data, it may be possible to achieve an automated regional outbreak detection system. However, privacy concerns and issues of transparency need to be addressed, and specificity and sensitivity need to be evaluated.

### Conclusion

Google search data may be useful in combination with clinical data to assist in estimating the incidence of infectious conjunctivitis in the context of epidemic outbreaks.

## Appendix

Multimedia Appendix 1 Population figures of the individual cities.

Multimedia Appendix 2 Conjunctivitis associated terms. All 714 associated terms were translated into English. In the German language, there are two terms for cataract, “Katarakt” and “grauer Star.” In addition, the term conjunctivitis is described with two words, “Konjunktivitis” and “Bindehautentzündung”.

## Abbreviations

ICD: International Statistical Classification of Diseases and Related Health Problems
