# Supplementary material for: Estimating the Incidence of Conjunctivitis by Comparing the Frequency of Google Search Terms With Clinical Data: Retrospective Study
Source: JMIR Public Health Surveill. 2021 Mar 3;7(3):e22645. doi: 10.2196/22645 (PMC7970297; doi:10.2196/22645)
Supplement: Multimedia Appendix 1 [file publichealth_v7i3e22645_app1.docx]

| **City** | **Population  (Record date 31.12.2017)** |
| --- | --- |
| Germany | 82,792,351 |
| Berlin | 3,613,495 |
| Hamburg | 1,830,584 |
| Munich | 1,456,039 |
| Cologne | 1,080,394 |
| Frankfurt (Main) | 746,878 |
| Stuttgart | 632,743 |
| Leipzig | 581,980 |
| Hannover | 535,061 |
| Nuremberg | 515,201 |
| Freiburg | 229,636 |
| Rostock | 208,409 |
| Kassel | 200,736 |
