# Supplementary material for: Estimating the Incidence of Conjunctivitis by Comparing the Frequency of Google Search Terms With Clinical Data: Retrospective Study
Source: JMIR Public Health Surveill. 2021 Mar 3;7(3):e22645. doi: 10.2196/22645 (PMC7970297; doi:10.2196/22645)
Supplement: Multimedia Appendix 2 [file publichealth_v7i3e22645_app2.docx]

| **German search term** | **English translation** |
| --- | --- |
| adenovirus konjunktivitis | adenovirus conjunctivitis |
| akupressur augenentzündung | acupressure eye inflammation |
| akute allergische konjunktivitis | acute allergic conjunctivitis |
| akute augenentzündung | acute eye inflammation |
| akute bindehautentzündung | acute conjunctivitis |
| akute konjunktivitis | acute conjunctivitis |
| allergie augenentzündung | allergy eye inflammation |
| allergie bindehautentzündung | allergy conjunctivitis |
| allergie oder bindehautentzündung | allergy or conjunctivitis |
| allergische augenentzündung | allergic eye inflammation |
| allergische bindehautentzündung | allergic conjunctivitis |
| allergische bindehautentzündung ansteckend | allergic conjunctivitis contagious |
| allergische bindehautentzündung augentropfen | allergic conjunctivitis eye drops |
| allergische bindehautentzündung kind | allergic conjunctivitis child |
| allergische bindehautentzündung nur ein auge | allergic conjunctivitis only one eye |
| allergische bindehautentzündung symptome | allergic conjunctivitis symptoms |
| allergische konjunktivitis behandlung | allergic conjunctivitis treatment |
| allergische konjunktivitis therapie | allergic conjunctivitis therapy |
| anfang bindehautentzündung | beginning of conjunctivitis |
| anfang bindehautentzündung kleinkind | beginning conjunctivitis infant |
| anfang einer bindehautentzündung | beginning of a conjunctivitis |
| anfängliche bindehautentzündung | initial conjunctivitis |
| ansteckende augenentzündung | contagious eye inflammation |
| ansteckende augeninfektion | contagious eye infection |
| ansteckende bindehautentzündung | infectious conjunctivitis |
| ansteckende bindehautentzündung kinder | infectious conjunctivitis children |
| ansteckungsgefahr bei bindehautentzündung | risk of infection with conjunctivitis |
| ansteckungsgefahr bindehautentzündung | danger of infection conjunctivitis |
| antibiotische augensalbe kinder | antibiotic eye ointment children |
| antibiotische augensalbe rezeptfrei | antibiotic eye ointment prescription free |
| antibiotische augensalbe rezeptpflichtig | antibiotic eye ointment on prescription only |
| antibiotische augentropfen | antibiotic eye drops |
| antibiotische augentropfen baby | antibiotic eye drops baby |
| antibiotische augentropfen bei bindehautentzündung | antibiotic eye drops for conjunctivitis |
| antibiotische augentropfen bindehautentzündung | antibiotic eye drops conjunctivitis |
| antibiotische augentropfen bindehautentzündung kind | antibiotic eye drops conjunctivitis child |
| antibiotische augentropfen bindehautentzündung rezeptfrei | antibiotic eye drops conjunctivitis prescription free |
| antibiotische augentropfen für kinder | antibiotic eye drops for children |
| antibiotische augentropfen kind | antibiotic eye drops child |
| antibiotische augentropfen kind rezeptfrei | antibiotic eye drop child prescription free |
| antibiotische augentropfen kleinkind | antibiotic eye drop infant |
| antibiotische augentropfen rezeptfrei | antibiotic eye drops prescription free |
| auge entzündet baby | eye inflamed baby |
| auge entzündet eiter | eye inflamed pus |
| auge entzündet kind | eye inflamed child |
| auge entzündet kontaktlinsen | eye inflamed contact lenses |
| augen bindehautentzündung | eyes conjunctivitis |
| augen bindehautentzündung symptome | eyes conjunctivitis symptoms |
| augen konjunktivitis | eyes conjunctivitis |
| augencreme bindehautentzündung | eye cream conjunctivitis |
| augenentzündung | eye inflammation |
| augenentzündung allergie | eye inflammation allergy |
| augenentzündung ansteckend | eye inflammation contagious |
| augenentzündung augentropfen | eye inflammation eye drops |
| augenentzündung aussen | eye inflammation outside |
| augenentzündung baby eiter | eye inflammation baby pus |
| augenentzündung bakteriell | bacterial eye inflammation |
| augenentzündung behandlung | eye inflammation treatment |
| augenentzündung bei erkältung | eye inflammation with cold |
| augenentzündung bei kindern | eye inflammation in children |
| augenentzündung bei meerschweinchen | eye inflammation in guinea pigs |
| augenentzündung beim hund | eye inflammation in dogs |
| augenentzündung bindehaut | eye inflammation conjunctiva |
| augenentzündung chronisch | chronic eye inflammation |
| augenentzündung durch erkältung | eye inflammation due to a cold |
| augenentzündung durch kontaktlinsen | eye inflammation from contact lenses |
| augenentzündung durch pilz | fungal eye inflammation |
| augenentzündung durch schnupfen | eye inflammation due to a cold |
| augenentzündung durch stress | eye inflammation due to stress |
| augenentzündung durch zugluft | eye inflammation due to draught |
| augenentzündung eiter | eye inflammation pus |
| augenentzündung eitrig | purulent eye inflammation |
| augenentzündung erkältung | eye inflammation cold |
| augenentzündung erwachsenen | eye inflammation adult |
| augenentzündung hamster | eye inflammation hamster |
| augenentzündung kind | eye inflammation child |
| augenentzündung kinder eiter | eye inflammation children pus |
| augenentzündung kleinkind | eye inflammation infant |
| augenentzündung kleinkind eiter | eye inflammation infant pus |
| augenentzündung kommt immer wieder | eye inflammation keeps coming back |
| augenentzündung konjunktivitis | eye inflammation conjunctivitis |
| augenentzündung kontaktlinsen | eye inflammation contact lenses |
| augenentzündung mensch | eye inflammation human |
| augenentzündung nach erkältung | eye inflammation after a cold |
| augenentzündung nach operation | eye inflammation after surgery |
| augenentzündung naturheilmittel | eye inflammation natural remedy |
| augenentzündung natürlich behandeln | treat eye inflammation naturally |
| augenentzündung neugeborenes | eye inflammation newborn |
| augenentzündung ohne rötung | eye inflammation without redness |
| augenentzündung schwangerschaft | eye inflammation pregnancy |
| augenentzündung stress | eye inflammation stress |
| augenentzündung symptome | eye inflammation symptoms |
| augenentzündung verklebte augen | eye inflammation glued eyes |
| augenentzündung virus | eye inflammation virus |
| augenentzündung zugluft | eye inflammation draught |
| augeninfektion | eye infection |
| augeninfektion ansteckend | eye infection contagious |
| augeninfektion kind | eye infection child |
| augeninfektion symptome | eye infection symptoms |
| augeninfektion virus | eye infection virus |
| augensalbe antibiotisch | eye ointment antibiotic |
| augensalbe antibiotisch rezeptfrei | eye ointment antibiotic prescription free |
| augensalbe bei bindehautentzündung | eye ointment for conjunctivitis |
| augensalbe bindehautentzündung | eye ointment conjunctivitis |
| augensalbe bindehautentzündung rezeptfrei | eye ointment conjunctivitis prescription free |
| augensalbe für bindehautentzündung | eye ointment for conjunctivitis |
| augensalbe gegen bindehautentzündung | eye ointment against conjunctivitis |
| augensalbe kinder bindehautentzündung | eye ointment children conjunctivitis |
| augensalbe kleinkind bindehautentzündung | eye ointment infant conjunctivitis |
| augensalbe konjunktivitis | eye ointment conjunctivitis |
| augensalbe rezeptfrei bindehautentzündung | eye ointment prescription free conjunctivitis |
| augentropfen allergische bindehautentzündung | eye drops allergic conjunctivitis |
| augentropfen antibiotisch | eye drops antibiotic |
| augentropfen baby bindehautentzündung | eye drops baby conjunctivitis |
| augentropfen bakterielle bindehautentzündung | eye drops bacterial conjunctivitis |
| augentropfen bakterielle infektion | eye drops bacterial infection |
| augentropfen bakterien | eye drops bacteria |
| augentropfen bei bakterieller bindehautentzündung | eye drops for bacterial conjunctivitis |
| augentropfen bei bindehautentzündung | eye drops for conjunctivitis |
| augentropfen bei bindehautentzündung kinder | eye drops for conjunctivitis children |
| augentropfen bei bindehautentzündung rezeptfrei | eye drops for conjunctivitis prescription free |
| augentropfen bei konjunktivitis | eye drops for conjunctivitis |
| augentropfen bindehaut | eye drop conjunctiva |
| augentropfen bindehautentzündung | eye drops conjunctivitis |
| augentropfen bindehautentzündung baby | eye drops conjunctivitis baby |
| augentropfen bindehautentzündung kind | eye drops conjunctivitis child |
| augentropfen bindehautentzündung kind rezeptfrei | eye drops conjunctivitis child prescription free |
| augentropfen bindehautentzündung kleinkind | eye drops conjunctivitis infant |
| augentropfen bindehautentzündung rezeptfrei | eye drops conjunctivitis prescription free |
| augentropfen bindehautentzündung rezeptfrei baby | eye drops conjunctivitis prescription free baby |
| augentropfen bindehautentzündung verschreibungspflichtig | eye drops conjunctivitis prescription only |
| augentropfen entzündete augen | eye drops inflamed eyes |
| augentropfen für baby bei bindehautentzündung | eye drops for baby with conjunctivitis |
| augentropfen für bindehautentzündung | eye drops for conjunctivitis |
| augentropfen für bindehautentzündung rezeptfrei | eye drops for conjunctivitis prescription free |
| augentropfen für kinder bei bindehautentzündung | eye drops for children with conjunctivitis |
| augentropfen für kinder bindehautentzündung | eye drops for children conjunctivitis |
| augentropfen für kinder gegen bindehautentzündung | eye drops for children against conjunctivitis |
| augentropfen gegen augenentzündung | eye drops against eye inflammation |
| augentropfen gegen bakterien | eye drops against bacteria |
| augentropfen gegen bindehautentzündung | eye drops against conjunctivitis |
| augentropfen gegen bindehautentzündung kinder | eye drops against conjunctivitis children |
| augentropfen gegen bindehautentzündung rezeptfrei | eye drops against conjunctivitis prescription free |
| augentropfen gegen entzündete augen | eye drops against inflamed eyes |
| augentropfen gegen konjunktivitis | eye drops against conjunctivitis |
| augentropfen gegen reizung | eye drops against irritation |
| augentropfen kinder antibiotisch | eye drops children antibiotic |
| augentropfen kinder bei bindehautentzündung | eye drops children with conjunctivitis |
| augentropfen kinder bindehautentzündung | eye drops children conjunctivitis |
| augentropfen kinder konjunktivitis | eye drops children conjunctivitis |
| augentropfen kleinkind bindehautentzündung | eye drops infant conjunctivitis |
| augentropfen konjunktivitis | eye drops conjunctivitis |
| augentropfen pflanzlich bindehautentzündung | herbal eye drops conjunctivitis |
| augentropfen rezeptfrei bindehautentzündung | eye drops prescription free conjunctivitis |
| augentrost baby | euphrasy baby |
| baby 3 monate bindehautentzündung | baby 3 months conjunctivitis |
| baby auge entzündet | baby eye inflamed |
| baby augenentzündung eiter | baby eye inflammation pus |
| baby augentropfen bindehautentzündung | baby eye drops conjunctivitis |
| baby bindehautentzündung | baby conjunctivitis |
| baby bindehautentzündung symptome | baby conjunctivitis symptoms |
| baby eiter auge | baby pus eye |
| baby eiter aus auge | baby pus from the eye |
| baby eiter im auge | baby pus in the eye |
| baby entzündete augen | baby inflamed eyes |
| baby gerötete augen | baby red eyes |
| baby konjunktivitis | baby conjunctivitis |
| baby ständig bindehautentzündung | baby constantly conjunctivitis |
| bakterielle augenentzündung | bacterial eye inflammation |
| bakterielle augenentzündung ansteckend | bacterial eye inflammation contagious |
| bakterielle augenentzündung kind | bacterial eye inflammation child |
| bakterielle augeninfektion | bacterial eye infection |
| bakterielle augeninfektion ansteckend | bacterial eye infection contagious |
| bakterielle bindehautentzündung | bacterial conjunctivitis |
| bakterielle bindehautentzündung ansteckend | bacterial conjunctivitis contagious |
| bakterielle bindehautentzündung augentropfen | bacterial conjunctivitis eye drops |
| bakterielle bindehautentzündung baby | bacterial conjunctivitis baby |
| bakterielle bindehautentzündung behandlung | bacterial conjunctivitis treatment |
| bakterielle bindehautentzündung kind | bacterial conjunctivitis child |
| bakterielle bindehautentzündung kinder | bacterial conjunctivitis children |
| bakterielle bindehautentzündung kleinkind | bacterial conjunctivitis infant |
| bakterielle bindehautentzündung symptome | bacterial conjunctivitis symptoms |
| bakterielle infektion am auge | bacterial infection of the eye |
| bakterielle infektion auge | bacterial infection eye |
| bakterielle infektion auge ansteckend | bacterial infection eye contagious |
| bakterielle infektion augen | bacterial infection eyes |
| bakterielle infektion im auge | bacterial infection in the eye |
| bakterielle konjunktivitis | bacterial conjunctivitis |
| bakterielle konjunktivitis kinder | bacterial conjunctivitis children |
| bakterielle konjunktivitis therapie | bacterial conjunctivitis therapy |
| bakterielle oder virale bindehautentzündung | bacterial or viral conjunctivitis |
| bakterien bindehautentzündung | bacteria conjunctivitis |
| bausch und lomb augentropfen bindehautentzündung | bausch and lomb eye drops conjunctivitis |
| beginn bindehautentzündung | beginning of conjunctivitis |
| beginn bindehautentzündung kind | beginning conjunctivitis child |
| beginn einer bindehautentzündung | beginning of a conjunctivitis |
| behandlung augenentzündung | treatment eye inflammation |
| behandlung bei bindehautentzündung | treatment for conjunctivitis |
| behandlung bindehautentzündung | treatment conjunctivitis |
| behandlung bindehautentzündung kinder | treatment conjunctivitis children |
| behandlung konjunktivitis | treatment conjunctivitis |
| behandlung virale bindehautentzündung | treatment of viral conjunctivitis |
| behandlung von bindehautentzündung | treatment of conjunctivitis |
| bei augenentzündung | during eye inflammation |
| bei bindehautentzündung | during conjunctivitis |
| beidseitige bindehautentzündung | bilateral conjunctivitis |
| berberil augentropfen bindehautentzündung | berberil eye drops conjunctivitis |
| berberil bei bindehautentzündung | berberil for conjunctivitis |
| berberil bindehautentzündung | berberil conjunctivitis |
| berberil n edo bindehautentzündung | berberil n edo conjunctivitis |
| binde augenentzündung | bandage eye inflammation |
| bindehaut | conjunctiva |
| bindehaut allergie | conjunctiva allergy |
| bindehaut am auge | conjunctiva of the eye |
| bindehaut ansteckend | conjunctiva contagious |
| bindehaut auge | conjunctiva eye |
| bindehaut augentropfen | conjunctiva eye drops |
| bindehaut baby | conjunctiva baby |
| bindehaut entzündet | inflamed conjunctiva |
| bindehaut im auge | conjunctiva in the eye |
| bindehaut kinder | conjunctiva children |
| bindehaut symptome | conjunctiva symptoms |
| bindehautent | conjunc |
| bindehautentz | conjunct |
| bindehautentzündung | conjunctivitis |
| bindehautentzündung 1 auge | conjunctivitis 1 eye |
| bindehautentzündung 1 jahr | conjunctivitis 1 year |
| bindehautentzündung allergie | conjunctivitis allergy |
| bindehautentzündung alternativ behandeln | treat conjunctivitis alternatively |
| bindehautentzündung am auge | conjunctivitis of the eye |
| bindehautentzündung an einem auge | conjunctivitis of one eye |
| bindehautentzündung anfang | conjunctivitis beginning |
| bindehautentzündung ansteckend | infectious conjunctivitis |
| bindehautentzündung ansteckend baby | conjunctivitis contagious baby |
| bindehautentzündung ansteckend hund | conjunctivitis contagious dog |
| bindehautentzündung ansteckungsgefahr | conjunctivitis risk of infection |
| bindehautentzündung antibiotische augentropfen | conjunctivitis antibiotic eye drops |
| bindehautentzündung apotheken umschau | conjunctivitis pharmacy magazine |
| bindehautentzündung arzt | conjunctivitis physician |
| bindehautentzündung auf beiden augen | conjunctivitis in both eyes |
| bindehautentzündung auf einem auge | conjunctivitis on one eye |
| bindehautentzündung auf kroatisch | conjunctivitis in croatian |
| bindehautentzündung auge | conjunctivitis eye |
| bindehautentzündung auge ansteckend | conjunctivitis eye contagious |
| bindehautentzündung auge symptome | conjunctivitis eye symptoms |
| bindehautentzündung augenlid | conjunctivitis eyelid |
| bindehautentzündung augensalbe | conjunctivitis eye ointment |
| bindehautentzündung augentropfen | conjunctivitis eye drops |
| bindehautentzündung augentropfen kinder | conjunctivitis eye drops children |
| bindehautentzündung augentropfen rezeptfrei | conjunctivitis eye drops prescription free |
| bindehautentzündung augentropfen verschreibungspflichtig | conjunctivitis eye drops prescription only |
| bindehautentzündung baby | conjunctivitis baby |
| bindehautentzündung baby 6 monate | conjunctivitis baby 6 months |
| bindehautentzündung baby ansteckend | conjunctivitis baby contagious |
| bindehautentzündung baby augentropfen | conjunctivitis baby eye drops |
| bindehautentzündung baby behandlung | conjunctivitis baby treatment |
| bindehautentzündung baby symptome | conjunctivitis baby symptoms |
| bindehautentzündung bakteriell | bacterial conjunctivitis |
| bindehautentzündung bakterien | conjunctivitis bacteria |
| bindehautentzündung beginn | conjunctivitis beginning |
| bindehautentzündung behandeln ohne arzt | treat conjunctivitis without a doctor |
| bindehautentzündung behandlung | conjunctivitis treatment |
| bindehautentzündung behandlung kinder | conjunctivitis treatment children |
| bindehautentzündung behandlung rezeptfrei | conjunctivitis treatment prescription free |
| bindehautentzündung bei erkältung | conjunctivitis during cold |
| bindehautentzündung bei erwachsenen | conjunctivitis at adults |
| bindehautentzündung bei grippe | conjunctivitis with flu |
| bindehautentzündung bei katzen | feline conjunctivitis |
| bindehautentzündung bei kindern | conjunctivitis in children |
| bindehautentzündung bei kindern behandeln | treat conjunctivitis in children |
| bindehautentzündung bei kindern symptome | conjunctivitis in children symptoms |
| bindehautentzündung bei neugeborenen | conjunctivitis in newborns |
| bindehautentzündung bei pferden | conjunctivitis on horses |
| bindehautentzündung bei säuglingen | conjunctivitis in newborns |
| bindehautentzündung bei schnupfen | conjunctivitis with cold |
| bindehautentzündung bei schwangeren | conjunctivitis in pregnant women |
| bindehautentzündung beide augen | conjunctivitis both eyes |
| bindehautentzündung beide augen behandeln | conjunctivitis both eyes treat |
| bindehautentzündung beidseitig | conjunctivitis on both sides |
| bindehautentzündung beim baby | conjunctivitis in the baby |
| bindehautentzündung beim kind | conjunctivitis in children |
| bindehautentzündung beim kleinkind | infant conjunctivitis |
| bindehautentzündung blind | conjunctivitis blind |
| bindehautentzündung blut | conjunctivitis blood |
| bindehautentzündung chronisch | conjunctivitis chronic |
| bindehautentzündung dexagent ophtal augensalbe | conjunctivitis dexagent ophthal ointment |
| bindehautentzündung dickes auge | conjunctivitis thick eye |
| bindehautentzündung doccheck | conjunctivitis doccheck |
| bindehautentzündung durch allergie | conjunctivitis due to allergy |
| bindehautentzündung durch erkältung | conjunctivitis due to cold |
| bindehautentzündung durch heuschnupfen | conjunctivitis due to hay fever |
| bindehautentzündung durch kontaktlinsen | conjunctivitis due to contact lenses |
| bindehautentzündung durch pollen | conjunctivitis due to pollen |
| bindehautentzündung durch schnupfen | conjunctivitis by cold |
| bindehautentzündung durch staub | conjunctivitis due to dust |
| bindehautentzündung durch stress | conjunctivitis due to stress |
| bindehautentzündung durch trockene augen | conjunctivitis due to dry eyes |
| bindehautentzündung durch viren | conjunctivitis caused by viruses |
| bindehautentzündung durch zugluft | conjunctivitis due to draught |
| bindehautentzündung durch zugluft ansteckend | conjunctivitis infectious by draught |
| bindehautentzündung durchfall | conjunctivitis diarrhea |
| bindehautentzündung ein auge | conjunctivitis one eye |
| bindehautentzündung eiter | conjunctivitis pus |
| bindehautentzündung eiter kind | conjunctivitis pus child |
| bindehautentzündung eitrig | conjunctivitis purulent |
| bindehautentzündung erbrechen | conjunctivitis vomit |
| bindehautentzündung erkältung | conjunctivitis colds |
| bindehautentzündung erste symptome | conjunctivitis first symptoms |
| bindehautentzündung erwachsene | adult conjunctivitis |
| bindehautentzündung erwachsene behandlung | conjunctivitis adult treatment |
| bindehautentzündung erwachsene symptome | conjunctivitis adult symptoms |
| bindehautentzündung gelbe augen | conjunctivitis yellow eyes |
| bindehautentzündung geschwollene augen | conjunctivitis swollen eyes |
| bindehautentzündung geschwollenes auge | conjunctivitis swollen eye |
| bindehautentzündung geschwollenes lid | conjunctivitis swollen lid |
| bindehautentzündung grippe | conjunctivitis flu |
| bindehautentzündung grüner schleim | conjunctivitis green mucus |
| bindehautentzündung heilt von alleine | conjunctivitis heals by itself |
| bindehautentzündung heuschnupfen | conjunctivitis hay fever |
| bindehautentzündung hiv | conjunctivitis hiv |
| bindehautentzündung hund ansteckend | conjunctivitis dog infectious |
| bindehautentzündung hund ansteckend für mensch | conjunctivitis dog contagious for human |
| bindehautentzündung husten | conjunctivitis cough |
| bindehautentzündung im auge | conjunctivitis in the eye |
| bindehautentzündung im urlaub | conjunctivitis on holiday |
| bindehautentzündung immer ansteckend | conjunctivitis always contagious |
| bindehautentzündung immer beide augen | conjunctivitis always both eyes |
| bindehautentzündung immer wieder | conjunctivitis over and over again |
| bindehautentzündung infektion | conjunctivitis infection |
| bindehautentzündung ist das ansteckend | conjunctivitis is this contagious |
| bindehautentzündung kind 1 jahr | conjunctivitis child 1 year |
| bindehautentzündung kind 2 jahre | conjunctivitis child 2 years |
| bindehautentzündung kind ansteckend für erwachsene | conjunctivitis child contagious for adults |
| bindehautentzündung kind antibiotische augentropfen | conjunctivitis child antibiotic eye drops |
| bindehautentzündung kind augentropfen | conjunctivitis child eye drops |
| bindehautentzündung kind eiter | conjunctivitis child pus |
| bindehautentzündung kinder | conjunctivitis children |
| bindehautentzündung kinder behandlung | conjunctivitis child treatment |
| bindehautentzündung kinder erkältung | conjunctivitis children colds |
| bindehautentzündung kinder kita | conjunctivitis children day-care center |
| bindehautentzündung kinder rezeptfrei | conjunctivitis children prescription free |
| bindehautentzündung kinder symptome | conjunctivitis children symptoms |
| bindehautentzündung kita | conjunctivitis day-care center |
| bindehautentzündung kleinkind | conjunctivitis infant |
| bindehautentzündung kleinkind ansteckend | conjunctivitis infant infectious |
| bindehautentzündung kleinkind augentropfen | conjunctivitis infant eye drops |
| bindehautentzündung kleinkind symptome | conjunctivitis infant symptoms |
| bindehautentzündung kochsalzlösung | conjunctivitis saline solution |
| bindehautentzündung kommt immer wieder | conjunctivitis comes again and again |
| bindehautentzündung kommt ständig wieder | conjunctivitis keeps coming back |
| bindehautentzündung kommt wieder | conjunctivitis comes back |
| bindehautentzündung konjunktivitis | conjunctivitis conjunctivitis |
| bindehautentzündung kontaktlinsen | conjunctivitis contact lenses |
| bindehautentzündung kroatisch | conjunctivitis croatian |
| bindehautentzündung latein | conjunctivitis latin |
| bindehautentzündung leicht | conjunctivitis light |
| bindehautentzündung mensch | conjunctivitis human |
| bindehautentzündung nach erkältung | conjunctivitis after cold |
| bindehautentzündung nach grippe | conjunctivitis after flu |
| bindehautentzündung nach katarakt op | conjunctivitis after cataract op |
| bindehautentzündung nach op | conjunctivitis after op |
| bindehautentzündung nach schwimmbad | conjunctivitis after swimming pool |
| bindehautentzündung nach star op | conjunctivitis after cataract op |
| bindehautentzündung naturheilkunde | conjunctivitis naturopathy |
| bindehautentzündung naturheilmittel | conjunctivitis natural remedy |
| bindehautentzündung natürlich behandeln | treat conjunctivitis naturally |
| bindehautentzündung natürlich heilen | conjunctivitis heal naturally |
| bindehautentzündung neugeborenes | conjunctivitis newborn |
| bindehautentzündung notdienst | conjunctivitis emergency service |
| bindehautentzündung nur an einem auge | conjunctivitis in one eye only |
| bindehautentzündung nur auf einem auge | conjunctivitis in one eye only |
| bindehautentzündung nur ein auge | conjunctivitis only one eye |
| bindehautentzündung oberes lid | conjunctivitis upper lid |
| bindehautentzündung oberlid | conjunctivitis upper eyelid |
| bindehautentzündung oder allergie | conjunctivitis or allergy |
| bindehautentzündung oder erkältung | conjunctivitis or cold |
| bindehautentzündung oder schnupfen | conjunctivitis or cold |
| bindehautentzündung ohne arzt | conjunctivitis without doctor |
| bindehautentzündung ohne behandlung | conjunctivitis without treatment |
| bindehautentzündung ohne eiter | conjunctivitis without pus |
| bindehautentzündung ohne rote augen | conjunctivitis without red eyes |
| bindehautentzündung ohne rotes auge | conjunctivitis without red eye |
| bindehautentzündung ohne rötung | conjunctivitis without redness |
| bindehautentzündung ohne verklebte augen | conjunctivitis without glued eyes |
| bindehautentzündung operation | conjunctivitis surgery |
| bindehautentzündung psyche | conjunctivitis psyche |
| bindehautentzündung rezeptfrei | conjunctivitis prescription free |
| bindehautentzündung rote augen | conjunctivitis red eyes |
| bindehautentzündung rotes auge | conjunctivitis red eye |
| bindehautentzündung samstag | conjunctivitis saturday |
| bindehautentzündung schleim | conjunctivitis mucus |
| bindehautentzündung schnell behandeln | treat conjunctivitis quickly |
| bindehautentzündung schnupfen | conjunctivitis cold |
| bindehautentzündung schulkind | conjunctivitis schoolchild |
| bindehautentzündung schwanger | conjunctivitis pregnant |
| bindehautentzündung schwangerschaft | conjunctivitis pregnancy |
| bindehautentzündung schwimmbad | conjunctivitis swimming pool |
| bindehautentzündung stress | conjunctivitis stress |
| bindehautentzündung symptome | conjunctivitis symptoms |
| bindehautentzündung symptome baby | conjunctivitis symptoms baby |
| bindehautentzündung symptome erwachsene | conjunctivitis symptoms adults |
| bindehautentzündung symptome kinder | conjunctivitis symptoms children |
| bindehautentzündung symptome kleinkind | conjunctivitis symptoms infant |
| bindehautentzündung therapie | conjunctivitis therapy |
| bindehautentzündung tränendes auge | conjunctivitis tearing eye |
| bindehautentzündung trockene augen | conjunctivitis dry eyes |
| bindehautentzündung und durchfall | conjunctivitis and diarrhoea |
| bindehautentzündung und erkältung | conjunctivitis and colds |
| bindehautentzündung und kontaktlinsen | conjunctivitis and contact lenses |
| bindehautentzündung und schnupfen | conjunctivitis and cold |
| bindehautentzündung und schwanger | conjunctivitis and pregnant |
| bindehautentzündung urlaub | conjunctivitis holiday |
| bindehautentzündung ventilator | conjunctivitis ventilator |
| bindehautentzündung verklebte augen | conjunctivitis glued eyes |
| bindehautentzündung verschreibungspflichtig | conjunctivitis prescription only |
| bindehautentzündung viren oder bakterien | conjunctivitis viruses or bacteria |
| bindehautentzündung virus | conjunctivitis virus |
| bindehautentzündung von innen nach aussen | conjunctivitis from inside to outside |
| bindehautentzündung wala | conjunctivitis wala |
| bindehautentzündung welche augentropfen | conjunctivitis which eye drops |
| bindehautentzündung weleda | conjunctivitis weleda |
| bindehautentzündung woher | conjunctivitis where from |
| bindehautentzündung woher kommt das | conjunctivitis where does it come from |
| bindehautentzündung zugluft | conjunctivitis draught |
| bindehautreizung | conjunctival irritation |
| bindehautreizung ansteckend | conjunctival irritation contagious |
| bindehautreizung augentropfen | conjunctival irritation eye drops |
| bindehautreizung kind | conjunctival irritation child |
| borwasser augenentzündung | boric acid solution eye inflammation |
| calendula augentropfen bindehautentzündung | calendula eye drops conjunctivitis |
| calendula bindehautentzündung | calendula conjunctivitis |
| chronisch entzündete augen | chronically inflamed eyes |
| chronische augenentzündung | chronic eye inflammation |
| chronische bindehautentzündung | chronic conjunctivitis |
| chronische bindehautentzündung hund | chronic conjunctivitis dog |
| chronische bindehautentzündung kind | chronic conjunctivitis child |
| chronische konjunktivitis | chronic conjunctivitis |
| corneregel bindehautentzündung | corneregel conjunctivitis |
| dauernd bindehautentzündung | permanent conjunctivitis |
| dermamycin augencreme bindehautentzündung | dermamycin eye cream conjunctivitis |
| dexa sine augentropfen bei bindehautentzündung | dexa sine eye drops for conjunctivitis |
| dexagent ophtal bindehautentzündung | dexagent ophtal conjunctivitis |
| dm augentropfen bindehautentzündung | dm eye drops conjunctivitis |
| echinacea augentropfen bindehautentzündung | echinacea eye drops conjunctivitis |
| ein auge entzündet | one eye inflamed |
| einseitige augenentzündung | unilateral eye inflammation |
| einseitige bindehautentzündung | unilateral conjunctivitis |
| einseitige konjunktivitis | unilateral conjunctivitis |
| eiter am auge | pus by the eye |
| eiter am auge baby | pus by the eye baby |
| eiter auge | purulent eye |
| eiter auge baby | pus eye baby |
| eiter aus auge | pus from the eye |
| eiter bindehautentzündung | pus conjunctivitis |
| eiter im auge | pus in the eye |
| eiter im auge baby | pus in the eye baby |
| c | pus in the eye cold |
| eiter im auge erkältung kleinkind | pus in the eye cold infant |
| eitrige augenentzündung | purulent eye inflammation |
| eitrige bindehautentzündung | purulent conjunctivitis |
| eitrige bindehautentzündung ansteckend | purulent conjunctivitis contagious |
| eitrige bindehautentzündung baby | purulent conjunctivitis baby |
| eitrige bindehautentzündung kind | purulent conjunctivitis child |
| eitrige bindehautentzündung kleinkind | purulent conjunctivitis infant |
| eitrige konjunktivitis | purulent conjunctivitis |
| eitrige konjunktivitis kinder | purulent conjunctivitis children |
| eitrige konjunktivitis therapie | purulent conjunctivitis therapy |
| entzündete augen | inflamed eyes |
| entzündete augen allergie | inflamed eyes allergy |
| entzündete augen baby | inflamed eyes baby |
| entzündete augen bei erkältung | inflamed eyes with cold |
| entzündete augen erkältung | inflamed eyes cold |
| entzündete augen kind | inflamed eyes child |
| entzündete augen kleinkind | inflamed eyes infant |
| entzündung bindehaut | inflammation conjunctiva |
| epidemische bindehautentzündung | epidemic conjunctivitis |
| epidemische konjunktivitis | epidemic conjunctivitis |
| erkältung auge entzündet | cold eye inflamed |
| erkältung augenentzündung | colds eye inflammation |
| erkältung bindehautentzündung | cold conjunctivitis |
| erkältung bindehautentzündung kind | cold conjunctivitis child |
| erkältung eiter auge | cold pus eye |
| erkältung eiter im auge | cold pus in the eye |
| erkältung entzündete augen | cold inflamed eyes |
| erkältung und augenentzündung | colds and eye inflammation |
| erkältung und bindehautentzündung | colds and conjunctivitis |
| erste symptome bindehautentzündung | first symptoms conjunctivitis |
| extreme bindehautentzündung | extreme conjunctivitis |
| follikuläre bindehautentzündung | follicular conjunctivitis |
| follikuläre konjunktivitis | follicular conjunctivitis |
| follikuläre konjunktivitis hund | follicular conjunctivitis dog |
| französische bulldogge augenentzündung | french bulldog eye inflammation |
| freiverkäufliche augentropfen bei bindehautentzündung | over-the-counter eye drops for conjunctivitis |
| freiverkäufliche augentropfen bindehautentzündung | over-the-counter eye drops conjunctivitis |
| freiverkäufliche augentropfen gegen bindehautentzündung | over-the-counter eye drops against conjunctivitis |
| frühjahrskatarrh | spring catarrh |
| gegen augenentzündung | against eye inflammation |
| gegen bindehautentzündung | against conjunctivitis |
| gegen bindehautentzündung kinder | against conjunctivitis children |
| gegen bindehautentzündung rezeptfrei | against conjunctivitis prescription free |
| gerötete augen baby | red eyes baby |
| gerötete augen bei kindern | reddened eyes in children |
| gerötete augen kind | reddened eyes child |
| gerötete augen kleinkind | red eyes toddler |
| geschwollene augen bindehautentzündung | swollen eyes conjunctivitis |
| geschwollene bindehaut | swollen conjunctiva |
| geschwollene verklebte augen | swollen glued eyes |
| geschwollenes auge bindehautentzündung | swollen eye conjunctivitis |
| gonokokken konjunktivitis | gonococcal conjunctivitis |
| grippe augenentzündung | flu eye inflammation |
| grippe bindehautentzündung | flu conjunctivitis |
| grippe und bindehautentzündung | flu and eye inflammation |
| h10 konjunktivitis | h10 conjunctivitis |
| habe ich eine bindehautentzündung | do I have a conjunctivitis |
| hamster augenentzündung | hamster eye inflammation |
| hartnäckige bindehautentzündung | persistent conjunctivitis |
| hase augenentzündung | rabbit eye inflammation |
| hase bindehautentzündung | rabbit conjunctivitis |
| häufige augenentzündung | frequent eye inflammation |
| häufige bindehautentzündung | frequent conjunctivitis |
| häufige bindehautentzündung bei erwachsenen | frequent conjunctivitis in adults |
| häufige bindehautentzündung bei kindern | frequent conjunctivitis in children |
| heilt bindehautentzündung von allein | cures conjunctivitis by itself |
| heuschnupfen bindehautentzündung | hay fever conjunctivitis |
| hiv bindehautentzündung | hiv conjunctivitis |
| hoch ansteckende augenentzündung | highly contagious eye inflammation |
| hoch ansteckende bindehautentzündung | highly contagious conjunctivitis |
| homöopathische augentropfen bei bindehautentzündung | homeopathic eye drops for conjunctivitis |
| homöopathische augentropfen bindehautentzündung | homeopathic eye drops conjunctivitis |
| hund bindehautentzündung ansteckend | dog conjunctivitis contagious |
| husten bindehautentzündung | cough conjunctivitis |
| hylo fresh bindehautentzündung | hylo fresh conjunctivitis |
| hylo parin bindehautentzündung | hylo parin conjunctivitis |
| icd konjunktivitis | icd conjunctivitis |
| immer entzündete augen | always inflamed eyes |
| immer wieder augenentzündung | recurrent eye inflammation |
| immer wieder bindehautentzündung | recurrent conjunctivitis |
| immer wieder bindehautentzündung kind | recurrent conjunctivitis child |
| immer wieder entzündete augen | frequently inflamed eyes |
| infectogenta augentropfen bindehautentzündung | infectogenta eye drops conjunctivitis |
| infektiöse augenentzündung | infectious eye inflammation |
| infektiöse bindehautentzündung | infectious conjunctivitis |
| infektiöse konjunktivitis | infectious conjunctivitis |
| iso augentropfen c bindehautentzündung | iso eye drops c conjunctivitis |
| ist bindehaut ansteckend | is conjunctiva contagious |
| ist bindehautentzündung ansteckend | is conjunctivitis contagious |
| ist bindehautentzündung ansteckend bei erwachsenen | is conjunctivitis contagious in adults |
| ist bindehautentzündung immer ansteckend | is conjunctivitis always contagious |
| ist eine augenentzündung ansteckend | is an eye inflammation contagious |
| ist eine bindehautentzündung ansteckend | is a conjunctivitis contagious |
| ist eine bindehautentzündung immer ansteckend | is a conjunctivitis always contagious |
| juckende augen bindehautentzündung | itchy eyes conjunctivitis |
| juckende verklebte augen | itchy glued eyes |
| kaninchen augenentzündung | rabbit eye inflammation |
| kann eine bindehautentzündung von alleine heilen | can a conjunctivitis heal on its own |
| katzen bindehautentzündung ansteckend | cats conjunctivitis contagious |
| kind 1 jahr bindehautentzündung | child 1 year conjunctivitis |
| kind auge entzündet | child eye inflamed |
| kind bindehautentzündung | child conjunctivitis |
| kind bindehautentzündung kita | child conjunctivitis day-care center |
| kind ständig bindehautentzündung | child permanently conjunctivitis |
| kinder augenentzündung | children eye inflammation |
| kinder augenentzündung eiter | children eye inflammation pus |
| kinder augentropfen bindehautentzündung | children eye drops conjunctivitis |
| kleinkind augenentzündung | Infant eye inflammation |
| kleinkind bindehautentzündung | infant conjunctivitis |
| kleinkind gerötete augen | infant reddened eyes |
| kleinkind rotes auge erkältung | infant red eye cold |
| kleinkind ständig bindehautentzündung | infant permanent conjunctivitis |
| kochsalzlösung bei bindehautentzündung | saline solution for conjunctivitis |
| kochsalzlösung bindehautentzündung | saline solution conjunctivitis |
| konjunktivitis | conjunctivitis |
| konjunktivitis ansteckend | conjunctivitis contagious |
| konjunktivitis ansteckungsgefahr | conjunctivitis risk of infection |
| konjunktivitis auge | conjunctivitis eye |
| konjunktivitis augentropfen | conjunctivitis eye drops |
| konjunktivitis baby | conjunctivitis baby |
| konjunktivitis bakteriell | bacterial conjunctivitis |
| konjunktivitis behandlung | conjunctivitis treatment |
| konjunktivitis bei erkältung | conjunctivitis with cold |
| konjunktivitis bei katzen | conjunctivitis in cats |
| konjunktivitis bei kindern | conjunctivitis in children |
| konjunktivitis hund therapie | conjunctivitis dog therapy |
| konjunktivitis kinder | conjunctivitis children |
| konjunktivitis kinder therapie | conjunctivitis children therapy |
| konjunktivitis kleinkind | infant conjunctivitis |
| konjunktivitis kleinkind therapie | conjunctivitis infant therapy |
| konjunktivitis kontaktlinsen | conjunctivitis contact lenses |
| konjunktivitis symptome | symptoms of conjunctivitis |
| konjunktivitis therapie | conjunctivitis therapy |
| konjunktivitis therapie kinder | conjunctivitis therapy children |
| kontaktlinsen bei bindehautentzündung | contact lenses for conjunctivitis |
| kontaktlinsen bindehautentzündung | contact lenses conjunctivitis |
| kontaktlinsen entzündete augen | contact lenses inflamed eyes |
| kontaktlinsen nach bindehautentzündung | contact lenses after conjunctivitis |
| leichte augenentzündung | slight eye inflammation |
| leichte bindehautentzündung | mild conjunctivitis |
| leichte bindehautentzündung kinder | slight conjunctivitis children |
| mechanische bindehautentzündung | mechanical conjunctivitis |
| mercurialis augentropfen bindehautentzündung | mercurialis eye drops conjunctivitis |
| mucokehl augentropfen bindehautentzündung | mucokehl eye drops conjunctivitis |
| nach bindehautentzündung | after conjunctivitis |
| nach bindehautentzündung trockene augen | after conjunctivitis dry eyes |
| nasentropfen bei bindehautentzündung | nasal drops for conjunctivitis |
| naturheilmittel bindehautentzündung | natural remedy conjunctivitis |
| neugeborenes augenentzündung | newborn eye inflammation |
| neugeborenes bindehautentzündung | newborn conjunctivitis |
| neugeborenes eiter im auge | newborn pus in the eye |
| oculoheel bindehautentzündung | oculoheel conjunctivitis |
| ofloxamed bindehautentzündung | ofloxamed conjunctivitis |
| oft bindehautentzündung | often conjunctivitis |
| pan ophtal augentropfen bindehautentzündung | pan ophtal eye drops conjunctivitis |
| pflanzliche augentropfen bindehautentzündung | vegetable eye drops conjunctivitis |
| ratiopharm augentropfen bindehautentzündung | ratiopharm eye drops conjunctivitis |
| rezeptfrei bindehautentzündung | prescription free conjunctivitis |
| rezeptfrei gegen bindehautentzündung | prescription free against conjunctivitis |
| rezeptfreie augentropfen bei bindehautentzündung | prescription free eye drops for conjunctivitis |
| rezeptfreie augentropfen bindehautentzündung | prescription free eye drops conjunctivitis |
| rezeptfreie augentropfen gegen bindehautentzündung | prescription free eye drops against conjunctivitis |
| rezidivierende bindehautentzündung | recurrent conjunctivitis |
| rezidivierende konjunktivitis | recurrent conjunctivitis |
| rki bindehautentzündung | rki conjunctivitis |
| rossmann augentropfen bindehautentzündung | rossmann eye drops conjunctivitis |
| rote augen bei erkältung kleinkind | red eyes during cold infant |
| rote augen bindehautentzündung | red eyes conjunctivitis |
| rote augen kinder erkältung | red eyes children cold |
| rote augen nach bindehautentzündung | red eyes after conjunctivitis |
| rote augen und eiter | red eyes and pus |
| rote eitrige augen | red purulent eyes |
| rote entzündete augen | red inflamed eyes |
| rote verklebte augen | red glued eyes |
| rote verklebte augen erkältung | red glued eyes cold |
| rotes auge bindehautentzündung | red eye conjunctivitis |
| rotes auge kind erkältung | red eye child cold |
| rotes auge und eiter | red eye and pus |
| scharlach augenentzündung | scarlet eye inflammation |
| schlimme bindehautentzündung | severe conjunctivitis |
| schnupfen augenentzündung | cold eye inflammation |
| schnupfen bindehautentzündung | cold conjunctivitis |
| schnupfen und bindehautentzündung | cold and conjunctivitis |
| schwanger bindehautentzündung | pregnant conjunctivitis |
| schwanger und bindehautentzündung | pregnant and conjunctivitis |
| schwangerschaft bindehautentzündung | pregnancy conjunctivitis |
| schwere augenentzündung | severe eye inflammation |
| schwere bindehautentzündung | severe conjunctivitis |
| schwimmbad bindehautentzündung | swimming pool conjunctivitis |
| silberwasser augenentzündung | silver water eye inflammation |
| silicea bindehautentzündung | silicea conjunctivitis |
| sinusitis bindehautentzündung | sinusitis conjunctivitis |
| ständig augenentzündung | persistent eye inflammation |
| ständig bindehautentzündung | persistent conjunctivitis |
| ständig bindehautentzündung kind | Permanent conjunctivitis child |
| ständig bindehautentzündung kleinkind | permanent conjunctivitis infant |
| starke augenentzündung | severe eye inflammation |
| starke bindehautentzündung | severe conjunctivitis |
| symptome augenentzündung | symptoms ophthalmitis |
| symptome bei bindehautentzündung | symptoms of conjunctivitis |
| symptome bindehautentzündung | symptoms conjunctivitis |
| symptome bindehautentzündung auge | symptoms conjunctivitis eye |
| symptome bindehautentzündung baby | symptoms conjunctivitis baby |
| symptome bindehautentzündung erwachsene | symptoms conjunctivitis adults |
| symptome bindehautentzündung kind | symptoms conjunctivitis child |
| symptome bindehautentzündung kleinkind | symptoms conjunctivitis infant |
| symptome einer bindehautentzündung | symptoms of conjunctivitis |
| symptome für bindehautentzündung | symptoms of conjunctivitis |
| symptome konjunktivitis | symptoms conjunctivitis |
| symptome von bindehautentzündung | symptoms of conjunctivitis |
| tetryzolin bindehautentzündung | tetryzolin conjunctivitis |
| therapie bei bindehautentzündung | therapy for conjunctivitis |
| therapie bindehautentzündung | therapy conjunctivitis |
| therapie konjunktivitis | therapy conjunctivitis |
| tränendes auge bindehautentzündung | teary eye conjunctivitis |
| trockene augen bindehautentzündung | dry eyes conjunctivitis |
| trockene augen nach bindehautentzündung | dry eyes after conjunctivitis |
| trockene augen oder bindehautentzündung | dry eyes or conjunctivitis |
| trockene bindehautentzündung | dry conjunctivitis |
| unspezifische bindehautentzündung | unspecific conjunctivitis |
| verklebte augen bindehautentzündung | glued eyes conjunctivitis |
| verklebte rote augen | glued red eyes |
| verschleppte bindehautentzündung | protracted conjunctivitis |
| virale augenentzündung | viral eye inflammation |
| virale augeninfektion | viral eye infection |
| virale bindehautentzündung | viral conjunctivitis |
| virale bindehautentzündung ansteckend | viral conjunctivitis contagious |
| virale bindehautentzündung baby | viral conjunctivitis baby |
| virale bindehautentzündung behandlung | viral conjunctivitis treatment |
| virale bindehautentzündung kind | viral conjunctivitis child |
| virale bindehautentzündung kleinkind | viral conjunctivitis infant |
| virale bindehautentzündung symptome | viral conjunctivitis symptoms |
| virale infektion auge | viral infection eye |
| virale konjunktivitis | viral conjunctivitis |
| virale konjunktivitis augentropfen | viral conjunctivitis eye drops |
| virale konjunktivitis kind | viral conjunctivitis child |
| virale konjunktivitis therapie | viral conjunctivitis therapy |
| virale oder bakterielle bindehautentzündung | viral or bacterial conjunctivitis |
| virus augenentzündung | virus eye inflammation |
| virus augeninfektion | virus eye infection |
| virus bindehautentzündung | virus conjunctivitis |
| virus konjunktivitis | virus conjunctivitis |
| vita pos augensalbe bindehautentzündung | vita pos eye ointment conjunctivitis |
| vividrin augentropfen bindehautentzündung | vivid eye drops conjunctivitis |
| vorbeugung bindehautentzündung | prevention conjunctivitis |
| wala augentropfen bindehautentzündung | wala eye drops conjunctivitis |
| wala bindehautentzündung | wala conjunctivitis |
| welche augensalbe bei bindehautentzündung | which eye ointment for conjunctivitis |
| welche augentropfen bei bindehautentzündung | which eye drops for conjunctivitis |
| welche augentropfen bei bindehautentzündung kind | which eye drops for conjunctivitis child |
| welche bindehautentzündung ist ansteckend | which conjunctivitis is contagious |
| weleda augentropfen bindehautentzündung | weleda eye drops conjunctivitis |
| wiederholte bindehautentzündung | recurrent conjunctivitis |
| windpocken augenentzündung | varicella eye inflammation |
| windpocken bindehautentzündung | varicella conjunctivitis |
| www bindehautentzündung auge | www conjunctivitis eye |
| yxin augentropfen bindehautentzündung | yxin eye drops conjunctivitis |
| zaditen ophtha sine bindehautentzündung | zaditen ophta sine conjunctivitis |
